# *Sanghuangporus vaninii* fruit body polysaccharide alleviates hyperglycemia and hyperlipidemia *via* modulating intestinal microflora in type 2 diabetic mice

**DOI:** 10.3389/fnut.2022.1013466

**Published:** 2022-10-17

**Authors:** Zi-Rui Huang, Qi-Zhen Huang, Ke-Wen Chen, Zi-Feng Huang, Yun Liu, Rui-Bo Jia, Bin Liu

**Affiliations:** ^1^College of Food Science, Fujian Agriculture and Forestry University, Fuzhou, China; ^2^Chaozhou Branch of Chemistry and Chemical Engineering Guangdong Laboratory, Chaozhou, China; ^3^National Engineering Research Center of JUNCAO Technology, Fujian Agriculture and Forestry University, Fuzhou, China

**Keywords:** *Sanghuangporus vaninii*, polysaccharide, hypoglycemic and hypolipidemic activity, inflammation, intestinal microflora

## Abstract

The disease of type 2 diabetes mellitus (T2DM) is principally induced by insufficient insulin secretion and insulin resistance. In the current study, *Sanghuangporus vaninii* fruit body polysaccharide (SVP) was prepared and structurally characterized. It was shown that the yield of SVP was 1.91%, and SVP mainly contains small molecular weight polysaccharides. Afterward, the hypoglycemic and hypolipidemic effects and the potential mechanism of SVP in T2DM mice were investigated. The results exhibited oral SVP could reverse the body weight loss, high levels of blood glucose, insulin resistance, hyperlipidemia, and inflammation in T2DM mice. Oral SVP increased fecal short-chain fatty acids (SCFAs) concentrations of T2DM mice. Additionally, 16S rRNA sequencing analysis illustrated that SVP can modulate the structure and function of intestinal microflora in T2DM mice, indicating as decreasing the levels of Firmicutes/Bacteroidetes, *Flavonifractor*, *Odoribacter*, and increasing the levels of *Weissella*, *Alloprevotella*, and *Dubosiella*. Additionally, the levels of predicted metabolic functions of Citrate cycle, GABAergic synapse, Insulin signaling pathway were increased, and those of Purine metabolism, Taurine and hypotaurine metabolism, and Starch and sucrose metabolism were decreased in intestinal microflora after SVP treatment. These findings demonstrate that SVP could potentially play hypoglycemic and hypolipidemic effects by regulating gut microflora and be a promising nutraceutical for ameliorating T2DM.

## Introduction

Diabetes mellitus is a metabolism disorder disease mainly characterized by high levels of blood glucose (BG) (1). According to the Diabetes Alliance survey, the adult diabetes rate in the world was as high as 8.3% in 2019, and this proportion will increase to 9.6% by 2045. Furthermore, type 2 diabetes mellitus (T2DM) has multifactorial and complex pathogenesis (2). The main hazard of T2DM is the long-term high BG, which leads to the occurrence of some chronic diseases and complications, including hyperlipidemia, inflammation, damage, and lesions of tissues and organs such as kidneys, eyeballs, hearts, and blood vessels (3). The existing strategy to alleviate diabetes mainly involves oral anti-hyperglycemic agents (biguanides, sulfonylureas, glucagon-like peptide-1 (GLP-1) receptor agonists, insulin sensitizers, α-glucosidase inhibitors, and thiazolidinedione) (4), which may be accompanied by many side effects (5). Therefore, it needs to be committed to developing novel antidiabetic drugs from natural sources with outstanding biological activity and low toxicity.

Growing evidences suggest that 16S rRNA sequencing has provided an important and effective platform to provide a standard method for studying intestinal microflora (6, 7). The interactions between the host and intestinal microflora powerfully affect the wellness and illness of the host. Hence, Dysbiosis or imbalance of the intestinal microflora is a hallmark of metabolic disease (8). Recently, fungal-derived polysaccharides have been shown to ameliorate metabolic syndrome by regulating intestinal microflora, such as *Ganoderma lucidum*, *Hericium erinaceus*, and *Grifola frondosa* (9). The treatment of diabetes by polysaccharides is associated with their fermentation by intestinal microflora to short-chain fatty acids (SCFAs), which play a key role in alleviating T2DM (10, 11).

“Sanghuang” is a popular medicinal fungus available, with a reputation of being “forest gold” in China and “Meshimakobu” in Japan (12). There are various confusing names for *Sanghuangporus*, *Phellinus linteus*, *Phellinus baumii*, *Inonotus sanghuang*, and “Sanghuang,” and others (13, 14). However, the three species of *Sanghuangporus sanghuang*, *Sanghuangporus baumii*, and *Sanghuangporus vaninii* were well-recognized in China (15). Recently, some modern pharmacological studies have illustrated “Sanghuang” has anti-tumor, hypoglycemic, antioxidant, and hepatoprotective effects (16). It was reported that *P. linteus* polysaccharide improves insulin signaling by modulating the ratio of phosphatidylcholine to phosphatidylethanolamine and the ratio of S-adenosylmethionine to S-adenosylhomocysteine in mouse plasma to lower BG concentrations, thereby increasing glucose tolerance ability (17). In addition, *P. linteus* water extract could decrease serum lipid by suppressing the activity of 3-hydroxy-3-methylglutaryl coenzyme-A coenzyme, which is a reductase in the liver (18). *P. linteus* polysaccharide extract could reduce lipopolysaccharide content, reduce inflammatory factors and reverse insulin resistance by regulating the abundance of intestinal microbiota that produces SCFAs (19). However, there is no research investigating the antidiabetic effects of *S. vaninii* fruit body polysaccharide (SVP).

Hence, this work was designed to prepare and characterize the SVP, and explore the underlying mechanism of oral SVP against T2DM in mice from the perspective of intestinal microflora. Moreover, the correlation between intestinal microflora, biochemical indexes, and SCFAs were investigated, providing a scientific reference for exploiting innovative active foods for people with T2DM-induced hyperglycemia and hyperlipidemia.

## Materials and methods

### Preparation of *Sanghuangporus vaninii* polysaccharide

Firstly, the dried powders of *S. vaninii* fruit body were treated with 30 times the volume of 70% (*v/v*) ethanol by ultrasound at 60°C for 1 h twice, to remove alcohol-soluble substances. Secondly, the residue was ultrasound (45 kHz, 300 W) with 50 times the volume of distilled water for 1 h at 60°C, and subsequently extracted by water for 2 h twice. Then, the combined supernatant was concentrated at 60°C and then added to four volumes of 95% (*v/v*) ethanol and kept at 4°C overnight. After centrifugation, the crude polysaccharide was collected and washed with ethanol, acetone, and ether. Next, the crude polysaccharide was further treated by the Sevag method to remove protein. Finally, the deproteinized supernatant was lyophilized to yield SVP after 48 h dialysis membranes (3.5 kDa cut-off), and the extraction yield of SVP was 1.91 ± 0.25%.

### Characterization analysis of *Sanghuangporus vaninii* fruit body polysaccharide

The total sugar content was measured using the phenol-sulfuric acid method, and the protein content was determined by Bradford’s method. The results showed that the total sugar and protein contents were 60.06 ± 1.67 and 3.41 ± 0.64%, respectively. Moreover, Monosaccharide composition analysis was analyzed using the 1-phenyl-3-methyl-5-pyrazolone (PMP) derivatization method according to the previous protocol (20). The average molecular weight of SVP was analyzed according to a previous study (21). For analysis of fourier transform-infrared (FT-IR) spectrometry, the dried SVP (0.5 mg) was mixed with KBr powder and pressed into a 1 mm pellet, then the characteristic organic functional groups (scan range: 4,000 to 370 cm^–1^) were analyzed by a Bruker VERTEX 33 spectrometer (Bruker Corporation, Germany) (22).

### Animals and diet

Forty male ICR mice (SPF, 4 weeks age) were sourced from Wu Shi’s Experimental Center Laboratory (Fuzhou, China). All mice were kept in clean conditions with a 12 h light/dark cycle and extensive ventilation, the temperature was set at 22–26°C, and the relative humidity was controlled at 50–60%. All mice were treated with a normal-fat diet (NFD) and water *ad libitum* in the first week. After that, the mice were randomly divided into two groups: (1) NC group: mice were fed a NFD for 4 weeks (*n* = 8); (2) HSHCD group: mice were fed a high-sucrose and high-cholesterol diet (15% lard, 15% sucrose, 1% cholesterol, 10% yolk, 0.2% sodium deoxycholate, and 58.8% NFD) for 4 weeks (*n* = 32). After 4 weeks of intervention, the HSHCD group mice were injected intraperitoneally with streptozotocin (STZ) solution at a dose of 45 mg/kg three times during 1 week. And the mice in the NC group were intraperitoneally injected with an equal volume of citric acid buffer solution. The fasting blood glucose (FBG) levels were detected by the BG meter, and the successfully modeled T2DM mice (FBG value >11.1 mmol/L) were selected for next experiment. Finally, the T2DM mice from HFD group were also randomly divided into four groups (*n* = 8): DC (diabetic control), MET (oral 100 mg/kg/day of metformin), SVP-L (oral 100 mg/kg/day of SVP), and SVP-H (oral 300 mg/kg/day of SVP). The body weight (BW) of each mouse was measured every 2 weeks. After the experiment, the feces of each mouse were collected into a special centrifuge tube, snap-frozen in liquid nitrogen and stored at −80°C. The blood of mice was collected by eyeball, and then centrifuged at 3,000 × *g* for 10 min to obtain serum and stored at −80°C.

### Oral glucose tolerance test

All animals fasted overnight, and the values of BG were detected and marked as BG_0 h_. Then, all the mice were immediately treated with oral administration of glucose solution (2 g/kg B.W.). Next, the BG concentrations of mice after 0.5, 1, and 2 h were also detected and marked as BG_0_._5 h_, BG_1 h_, and BG_2 h_, respectively. Finally, the area under the curve (AUC) were calculated by formula: AUC = 0.5 × (0.5 × BG_0 h_ + BG_0_._5 h_ + 1.5 × BG_1 h_ + BG_2 h_).

### Determination of biochemical indexes

The levels of total cholesterol (TC), triglyceride (TG), high-density lipoprotein-cholesterol (HDL-c), low-density lipoprotein-cholesterol (LDL-c), free fatty acid (FFA), total bile acid (TBA), and glycated serum protein (GSP) were determined by assay kits (Jiancheng, Nanjing, China). In addition, fasting insulin (FINS), GLP-1, tumor necrosis factor-α (TNF-α), interleukin-6 (IL-6), and interleukin-10 (IL-10) levels were determined by ELISA kits (Chundu, Wuhan, China). Besides, the levels of insulin-related indexes were also calculated by formulas: HOMA-IRI = FBG (mmol/L) × FINS (mIU/L)/22.5; HOMA-ISI = 1/[FBG (mmol/L) × FINS (mIU/L)].

### Measurement of short-chain fatty acids

The fecal sample of each mouse (250 mg) was put into a 2 ml centrifuge tube with 1 ml ultra-pure water. The mixture was centrifuged (4°C, 135,399 × *g*) for 5 min, and then the supernatant was added to 0.1 ml 50% concentrated sulfuric acid (*v/v*) and 1 ml anhydrous ether. The mixture was centrifuged (4°C, 135,399 × *g*) for 10 min. The sample was prepared by taking the ether layer with a 0.22 μm filter membrane. Different concentrations of SCFAs standard products were prepared. Then the GC-MS program was conducted according to a previous study (23).

### Sequencing of intestinal microflora

The DNA genome was extracted from feces, and the V3–V4 region of the bacterial 16S rRNA was amplified with universal primers 338F and 806R. The 16S rRNA gene sequencing libraries of bacteria were produced by using a TruSeq DNA PCR-Free Sample Preparation Kit (Illumina, San Diego, CA, USA). The raw reads were obtained from Illumina NovaSeq PE250 platform (24), and the high-throughput sequencing was conducted by Novogene Co., Ltd. (Beijing, China).

### Analysis of intestinal microflora

Principal component analysis (PCA) in different groups was performed using SIMCA-14.1 (UMETRICS, Sweden). The differential abundances of intestinal microflora between paired groups using STAMP 2.1.3. The correlation data were analyzed and visualized as heatmap by R 4.1.0 with packages of “psych” and “pheatmap.” The correlation network was visualized by Cytoscape 3.9.1.

### Statistical analysis

The results of this work were presented as mean ± standard deviation (SD), and the GraphPad Prism 8.4 was used for the statistical significance of one-way ANOVA analysis with Turkey’s test, and the significance level was set at *p* < 0.05 and marked with different letters.

## Results and discussion

### Characterization of *Sanghuangporus vaninii* fruit body polysaccharide

**FIGURE 1:**
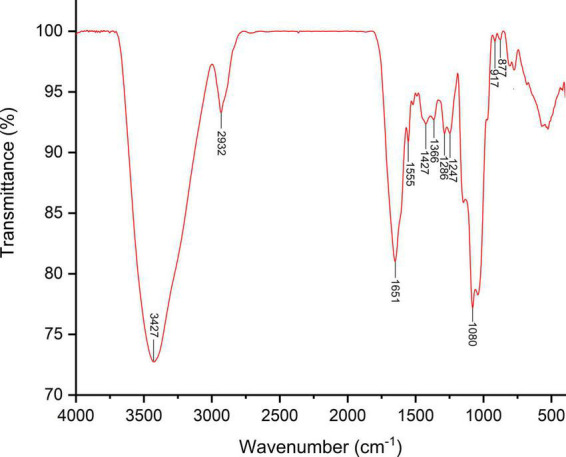
Fourier transform-infrared spectra of SVP.

The bioactivities of polysaccharides are hugely influenced by their chemical composition and structure characteristics (25). The results showed SVP is mainly composed of mannose, rhamnose, glucuronic acid, galacturonic acid, galactose, and arabinose in a molar ratio of 35.23, 6.45, 2.33, 9.64, 16.13, and 28.94%, respectively (Supplementary Table 1). The results of average molecular weight showed that SVP were mainly composed of 4 peaks, and the mass fractions of peak 1, 2, 3, and 4 were 4.7% (Mw 1051.4 kDa), 65.8% (Mw 31.2 kDa), 12.5% (Mw 12.5 kDa), and 4.9% (Mw 12.3 kDa), respectively (Supplementary Figure 1 and Supplementary Table 2). The above results exhibited that the main components of SVP are small molecular weight polysaccharides. According to several studies in recent years, low molecular weight polysaccharides usually have higher antioxidant (26) and antitumors (27), hypoglycemic activities (28), and potential as prebiotics (29). FT-IR spectroscopy is a powerful technique for the identification of characteristic organic groups in the bioactive polysaccharide and proteins. As shown in Figure 1, the strong absorption peak at 3,427 cm^–1^ was attributed to the tensile vibration of O-H. The small absorption peak at 2,932 cm^–1^ represents the stretching and bending vibrations of C-H (22). The two absorption peaks at 1,651 and 1,555 cm^–1^ are related to the stretching vibrations of C=O. The absorption peaks at 1,427 and 1,366 cm^–1^ accorded with the stretching and bending vibrations of C-H. The absorption peaks at 1,286 and 1,247 cm^–1^ were associated with the asymmetric O=S=O. The strong absorption peak at 1,080 cm^–1^ indicates the stretching vibration of C-O-C (21). The stretching vibration at 917–877 cm^–1^ were related to both α-glycosidic and β-glycosidic bonds between glucosamine units.

### Effects of oral *Sanghuangporus vaninii* fruit body polysaccharide on body weight and fasting blood glucose

**FIGURE 2:**
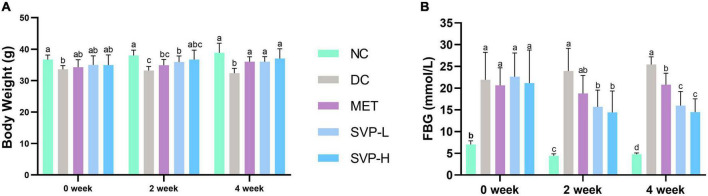
Effects of oral SVP on the body and blood glucose-related indexes in T2DM mice at 0th, 2nd, and 4th week. **(A)** Body weight levels; **(B)** fasting blood glucose levels. Different superscript letters indicate statistically significant differences between the groups (*p* < 0.05).

Body weight (BW) loss is one of the typical symptoms of STZ-injected diabetic mice (30). Compared with NC group, a significant (*p* < 0.01) weight loss of mice in DC group was observed from 0 to 4 weeks of the experiment (Figure 2A). After 2 weeks of intervention, SVP-L supplementation strongly (*p* < 0.01) reversed the weight loss in T2DM mice. However, BW of MET and SVP-H group mice did not show statistical differences from DC group. Compared with DC group, BW of MET, SVP-L, and SVP-H group mice were significantly (*p* < 0.01) increased at fourth week of experiment. These results suggested that long-term administration of SVP could improve impaired energy metabolism, carbohydrate utilization, and the symptom of weight loss in T2DM mice. It was illustrated FBG values of T2DM mice were markedly (*p* < 0.01) higher than that of NC group mice at initial stage of experiment (Figure 2B). At second week of intervention, except for MET group, the SVP intervention significantly (*p* < 0.05) reduced the FBG values in contrast to DC group mice. In addition, oral administration of metformin, SVP-L, and SVP-H for 4 weeks significantly (*p* < 0.01) decreased the FBG levels of T2DM mice by 18.33, 37.30, and 43.15%, respectively. It was demonstrated that SVP could regulate BG with a dose-dependent effect.

### Hypoglycemic and hypolipidemic effects of oral *Sanghuangporus vaninii* fruit body polysaccharide

**FIGURE 3:**
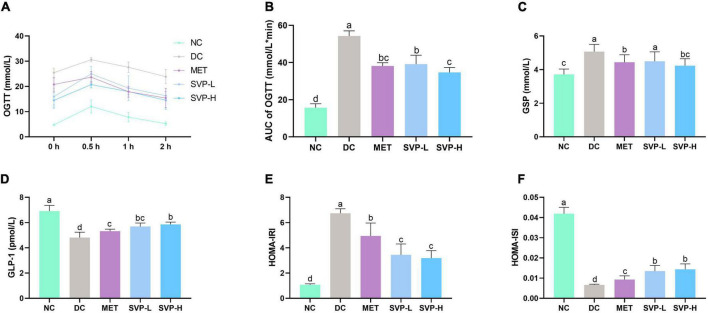
Effects of oral SVP on the glucose tolerance and islet function-related indexes in T2DM mice. **(A)** Oral glucose tolerance test levels; **(B)** area under the curve values of oral glucose tolerance test; **(C)** glycated serum protein levels; **(D)** glucagon-like peptide-1 levels; **(E)** HOMA-insulin resistance index levels; **(F)** HOMA-insulin sensitivity index levels. Different superscript letters indicate statistically significant differences between the groups (*p* < 0.05).

Oral glucose tolerance test (OGTT) is often used to assess the ability of glucose toleration in human or experimental animals. Generally, the BG concentration will be maintained in a relatively stable range, indicating that the body has a strong tolerance (31). The BG levels in each group increased and peaked at 0.5 h, then gradually decreased (Figure 3A). In comparison with NC group, the AUC values were strongly (*p* < 0.001) elevated by 246.06% in DC group (Figure 3B), suggesting glucose tolerance of HFD rats was impaired. In comparison with DC group, the AUC values of MET, SVP-L and SVP-H group mice were all significantly decreased (*p* < 0.001), and the reduction ratios were 29.74, 27.93, and 36.14%, respectively. The experiment showed that SVP intervention decreased the levels of AUC of OGTT in a dose-addicted manner, and could partly restore the glucose tolerance in T2DM mice. GSP is a delicate indicator of diabetes, which is not affected by the fluctuation of interim BG concentration and could provide feedback on the brief-term remedial effect of the drug (32). GSP level in DC group was significantly (*p* < 0.001) increased compared with NC group (Figure 3C). By contrast, MET, SVP-L, and SVP-H intervention for 4 weeks significantly (*p* < 0.05) decreased the GSP levels by 12.55, 11.42, and 16.57%, respectively. GLP-1 is not only a gastrointestinal hormone but also a neurotransmitter, and it was used to combat T2DM as a new therapeutic (33). As shown in Figure 3D, a significant loss of GLP-1 concentration was found in DC group compared with NC group. In contrast to DC group, MET, SVP-L, and SVP-H intervention for 4 weeks all significantly elevated the GLP-1 concentrations in T2DM mice. In short, it can be speculated that SVP has a similar hypoglycemic ability with metformin.

The HOMA of β-cell function (HOMA-β) was developed to quantize basic insulin secretion. And the increased HOMA-IRI level and decreased HOMA-ISI level are the primary characteristics of T2DM (22). Compared with NC group, the HOMA-IRI level in DC group was significantly (*p* < 0.001) increased whereas the HOMA-ISI level in DC group was significantly (*p* < 0.001) decreased (Figures 3E,F), suggesting that DC group mice had severe insulin resistance and lower insulin sensitivity. Moreover, compared with DC group, levels of HOMA-IRI in SVP-L and SVP-H groups were obviously (*p* < 0.001) decreased by 48.82 and 52.69%, respectively. At the same time, SVP-L and SVP-H significantly increased (*p* < 0.001) the values of HOMA-ISI by 103.84 and 117.29%, respectively, which showed a dose-dependence. These results suggest that SVP treatment could partly improve the insulin resistance and restore the insulin sensitivity in T2DM mice.

**FIGURE 4:**
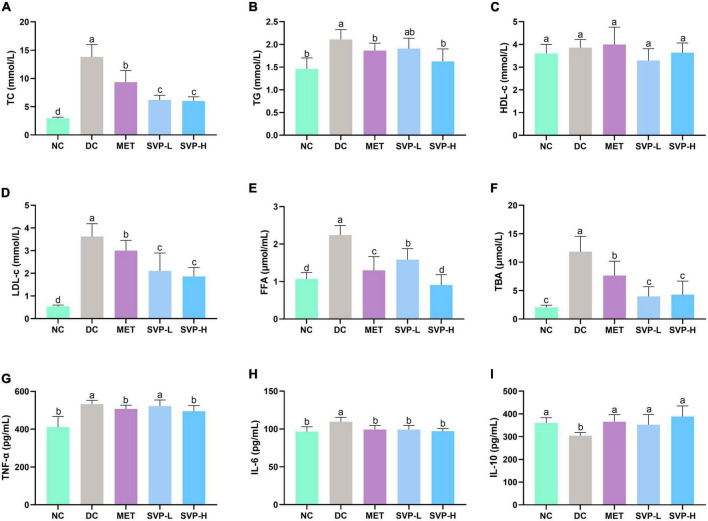
Effects of oral SVP on the lipid metabolism and inflammation-related indexes in T2DM mice. **(A)** Total cholesterol levels; **(B)** total triglyceride levels; **(C)** high-density lipoprotein-cholesterol levels; **(D)** low-density lipoprotein-cholesterol levels; **(E)** free fatty acid levels; **(F)** total bile acid levels; **(G)** tumor necrosis factor-α; **(H)** interleukin-6 levels; **(I)** interleukin-10 levels. Different superscript letters indicate statistically significant differences between the groups (*p* < 0.05).

The long-term hyperglycemia in diabetic patients will inevitably cause damage to various organs, and further lead to a series of chronic complications, especially the hyperlipidemia (2, 21). The concentrations of TC, TG, LDL-c, FFA, and TBA in serum were all significantly (*p* < 0.001) higher in DC group mice than those in NC group mice (Figures 4A–F). After 4 weeks of experiment, MET and SVP intervention significantly decreased the above lipid biochemical indexes. Especially, SVP-H intervention reduced TC, TG, LDL-c, FFA, and TBA levels by 56.49% (*p* < 0.001), 22.96% (*p* < 0.01), 48.68% (*p* < 0.001), 59.42% (*p* < 0.001), and 63.69% (*p* < 0.001), respectively. Interestingly, in contrast to DC group, there were no statistical differences of serum HDL-c levels in NC, MET, SVP-L, and SVP-H groups, which is consistent with a previous study (34). In short, it was indicated that SVP could prevent T2DM-induced hyperlipidemia, thereby reducing the risk of diabetic complications (35).

### Anti-inflammation effects of oral *Sanghuangporus vaninii* fruit body polysaccharide

Chronic low-grade inflammation often occurs in patients with islet impairment and T2DM (36). Abnormally increased or decreased inflammatory cytokines could lead to islet dysfunction in diabetic patients (37). Compared with NC group, the serum concentrations of both TNF-α and IL-6 were obviously (*p* < 0.001) increased (Figures 4G,H) in DC group, which is consistent with a previous report (38). However, the serum IL-10 concentration was significantly (*p* < 0.05) decreased (Figure 4I) in DC group. In contrast to DC group, decreased TNF-α levels were observed in MET (4.70%, *p* < 0.05), SVP-L (1.91%), and SVP-H (6.98%, *p* < 0.05) groups, and the reduced IL-6 levels were found in MET (9.40%, *p* < 0.01), SVP-L (9.48%, *p* < 0.01), and SVP-H (11.38%, *p* < 0.001) groups. On the contrary, serum IL-10 level was significantly increased by 20.12% (*p* < 0.01), 15.80% (*p* < 0.05), and 27.53% (*p* < 0.001) in MET, SVP-L, and SVP-H groups, respectively. The results demonstrated that SVP intervention could ameliorated the inflammation status in T2DM mice.

### Effects of oral *Sanghuangporus vaninii* fruit body polysaccharide on fecal short-chain fatty acids concentrations

**FIGURE 5:**
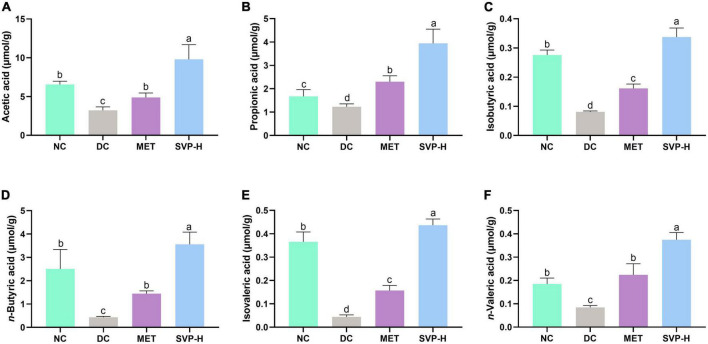
Effects of oral SVP on the levels of fecal short-chain fatty acids (SCFAs). **(A)** Acetic acid; **(B)** propionic acid; **(C)** isobutyric acid; **(D)** *n*-butyric; **(E)** isovaleric acid; **(F)** *n*-valeric acid. Different superscript letters indicate statistically significant differences between the groups (*p* < 0.05).

The advantage of polysaccharides in improving diabetes is that they are fermented into SCFAs by intestinal microflora, the lack of which may lead to further development of T2DM (39). Compared to NC group mice, the concentrations of acetic acid, propionic acid, isobutyric acid, *n*-butyric acid, isovaleric acid, and *n*-valeric acid in DC group were strongly reduced by 50.76% (*p* < 0.001), 26.47% (*p* < 0.05), 70.60% (*p* < 0.001), 82.73% (*p* < 0.001), 87.89% (*p* < 0.001), and 54.21% (*p* < 0.001) (Figure 5). After oral administrated by SVP-H for 4 weeks, the SCFAs concentrations were significantly (*p* < 0.001) increased by 203.83, 220.56, 315.82, 723.30, 886.77, and 342.76%, respectively. The results demonstrated oral SVP can markedly enhance the SCFAs production by intestinal microflora *in vivo*.

### Effects of oral *Sanghuangporus vaninii* fruit body polysaccharide on the structure of intestinal microflora

**FIGURE 6:**
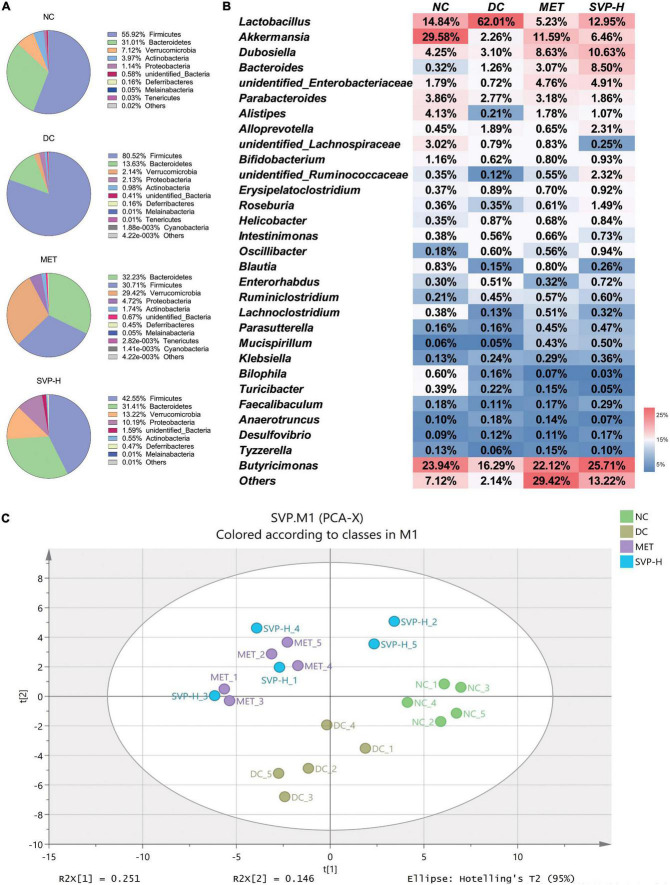
Effects of oral SVP on the relative abundances and the structure of intestinal microbiota in T2DM mice. **(A)** Components of intestinal microbiota at phylum levels; **(B)** components of intestinal microbiota at genus levels; **(C)** principle component analysis score plot of intestinal microbiota at genus levels.

Recently, intestinal microflora has received great attention, and its structure and stability are important for the maintenance of intestinal balance (40). Studies have shown that altering specific microorganisms in the intestine can affect the development of diabetes (41, 42). In this study, 16S rRNA based sequencing analysis was conducted to investigate the effects of SVP on the structure of intestinal microflora. It was illustrated that the phylum level of microflora was dominated by Firmicutes, Bacteroidetes, Verrucomicrobia, Actinobacteria, Proteobacteria, etc. (Figure 6A). Obviously, the Firmicutes population was increased from 55.92% in NC group to 80.52% in DC group. Simultaneously, the relative abundance of Bacteroidetes was reduced from 31.01 to 13.63% in DC group mice. However, the Firmicutes abundances of MET and SVP-H group were decreased to 30.71 and 42.55%, respectively, whereas the Bacteroidetes abundances of MET and SVP-H group were increased to 32.23 and 31.41%. Afterward, the Firmicutes/Bacteroidetes (F/B) ratio in DC group (5.91) was strongly higher than that of NC group (1.80). After treatment with metformin and SVP, the F/B values were decreased to 0.95 and 1.35, respectively. Usually, the F/B value is used as an essential indicator of the degree of intestinal microflora disturbance, and it is positively related hyperglycemia and hyperlipidemia in many studies (43–45). As shown in Figure 6B, the *Lactobacillus*, *Akkermansia*, *Dubosiella*, *Bacteroides*, *unidentified_Enterobacteriaceae, Parabacteroides*, and other genera dominated the fecal microorganisms. Compared with NC group, *Lactobacillus* population in DC group was strongly elevated, whereas the relative abundances of *Akkermansia*, *Dubosiella*, *Alistipes*, and others were decreased. In contrast to DC group, *Lactobacillus* population in the MET and SVP-H groups were obviously decreased. By contrast, *Akkermansia*, *Dubosiella*, *Bacteroides*, and other genera abundances were significantly increased. In short, these findings revealed that oral SVP could alter the T2DM-induced imbalanced structure of intestinal microflora at phylum and genus levels.

The α-diversity analysis demonstrated that the SVP-H intervention could partly reverse the reduction of Shannon, Simpson, Chao1, and ACE indexes of intestinal microflora in T2DM mice (Supplementary Table 3). Furthermore, β-diversity is the difference in diversities across samples or environments, which indicated differences in intestinal microflora composition in this study (46). PCA is an effective indicator for evaluating β-diversity (47). It was illustrated that an obvious separation between NC and DC groups (Figure 6C), indicating T2DM could induce huge structural changes of intestinal microflora. In detail, the symbols of NC group are mainly distributed beside the positive *x*-axis whereas those of DC group are mainly enriched beside the negative *y*-axis. Interestingly, the symbols of MET and SVP-H group were mainly distributed at the first and second quadrant, which have great distances from NC group and DC group. It was indicated that SVP had the potential to alleviate T2DM by changing the composition of intestinal microflora to restore gut dysbiosis.

### Effects of oral *Sanghuangporus vaninii* fruit body polysaccharide on the metabolic function of intestinal microflora

**FIGURE 7:**
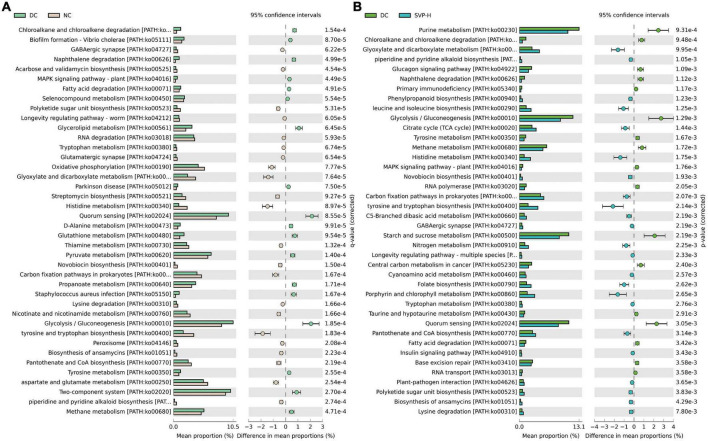
The differences of predicted function of the intestinal microbiota based on PICRUSt2 with Welch’s *t*-test and the *p*-value corrected by Benjamini–Hochberg method. **(A)** DC group versus NC group; **(B)** DC group versus SVP-H group. Corrected *p* < 0.05, confidence intervals = 95%, top 40 functional modules are presented according to the order of corrected *p*-value.

Furthermore, using PICRUSt2 to predict the functional potential of microbial communities through marker gene sequencing maps is a common strategy in amplicon sequencing (48). Compared to the previous version, PICRUSt2 provides a more credible algorithm to explore predicted metabolic function modules in intestinal microflora. Through the KEGG database’s comparison and analysis, the intestinal microbiota’s metabolic function in different groups was analyzed (49). Compared with NC group, there are 18 up-regulated functional modules in DC group (Figure 7A), such as Fatty acid degradation [PATH:ko00071], Quorum sensing [PATH:ko02024], D-Alanine metabolism [PATH:ko00480], Glycerolipid metabolism [PATH:ko00561], Glycolysis/Gluconeogenesis [PATH:ko00010], etc. By contrast, 22 functional modules of GABAergic synapse [PATH:ko04727], Longevity regulating pathway-worm [PATH:ko04212], Oxidative phosphorylation [PATH:ko00190], Histidine metabolism [PATH:ko00340], Thiamine metabolism [PATH:ko00730], etc. were reduced. Compared with DC group, there are 22 up-regulated functional modules of the top 40 modules in MET group, such as Arginine and proline metabolism [PATH:ko00330], GABAergic synapse [PATH:ko04727], Flote biosynthesis [PATH:ko00790], Citrate cycle (TCA cycle) [PATH:ko00020], etc. and the down-regulated functional modules contains Purine metabolism [PATH:ko00230], Quorum sensing [PATH:ko02024], Glycerolipid metabolism [PATH:ko00561], Starch and sucrose metabolism [PATH:ko00500], and so on (Supplementary Figure 2). As shown in Figure 7B, the 23 up-regulated and 17 down-regulated functional modules were illustrated in SVP-H group in contrast to DC group. For instance, the modules of Citrate cycle (TCA cycle) [PATH:ko00020], GABAergic synapse [PATH:ko04727], Histidine metabolism [PATH:ko00340], Longevity regulating pathway-multiple species [PATH:ko04213], Insulin signaling pathway [PATH:ko04910] were increased whereas Purine metabolism [PATH:ko00230], Glycolysis/Gluconeogenesis [PATH:ko00010], Fatty acid degradation [PATH:ko00071], Taurine and hypotaurine metabolism [PATH:ko00430], Starch and sucrose metabolism [PATH:ko00500] were decreased after SVP intervention in intestinal microflora of T2DM mice, which are similar to the effects of MET intervention in this study. These results provide a novel insight into the beneficial effects of SVP intervention on the T2DM-induced imbalance of microbial function.

### Correlations of the characteristic intestinal microbes and biochemical indexes

**FIGURE 8:**
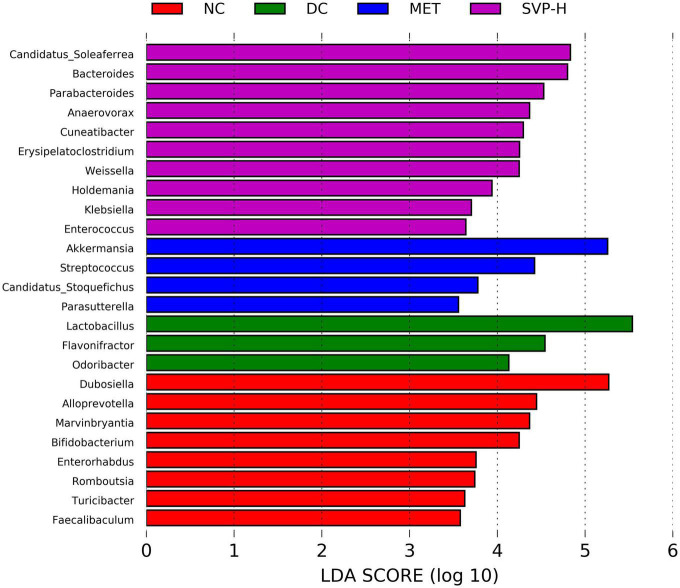
The characteristic intestinal microbes at genus level among NC, DC, MET, and SVP-H groups conducted by LEfSe analysis. LDA > 3.0, *p* < 0.05.

**FIGURE 9:**
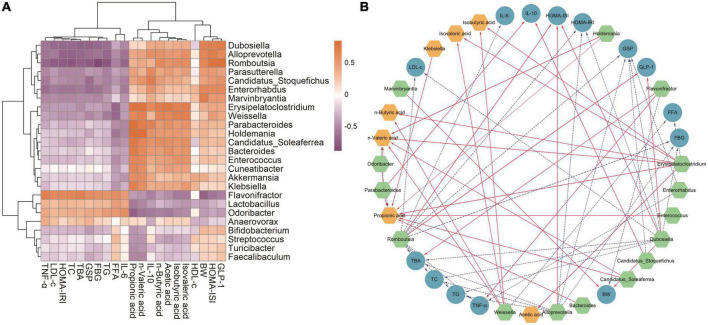
Statistical Spearman’s correlations between the characteristic intestinal microbes and the biochemical indexes in T2DM mice. **(A)** Hierarchical clustering heatmap of the correlation between the characteristic intestinal microbes and the biochemical indexes. The chocolate and orchid nodes represent the positive and negative correlation coefficient, respectively; the color shades indicate the strength of correlation. **(B)** Visualization of the correlation network. Green, blue, and yellow nodes represent the characteristic intestinal microbes, biochemical indexes, and fecal SCFAs, respectively; the solid red lines and dotted blue lines indicate the correlation coefficient >0.6 (*p* < 0.01) and the correlation coefficient <–0.6 (*p* < 0.01), respectively. The linewidth reflects on the strength of correlation.

Linear discriminant analysis (LDA) at the genus level was applied to further explore the effects of SVP intervention on the characteristic intestinal microbes in NC, DC, MET, and SVP-H groups. As shown in Figure 8, total of 25 characteristic microbes were analyzed with a threshold of LDA >3 and *p* < 0.05 among NC, DC, MET, and SVP-H groups. In detail, there were eight characteristic microbes enriched in NC group, namely *Dubosiella*, *Alloprevotella*, *Marvinbryantia*, *Bifidobacterium*, *Enterorhabdus*, *Romboutsia*, *Turicibacter*, and *Faecalibaculum*. In addition, three characteristic microbes were enriched in DC group, namely *Lactobacillus*, *Flavonifractor*, and *Odoribacter*. After metformin treatment, *Akkermansia*, *Streptococcus*, *Candidatus_Stoquefichus*, and *Parasutterella* populations were significantly increased in T2DM mice. Afterward, SVP-H group has 10 characteristic microbes, including *Candidatus_Soleaferrea*, *Bacteroides*, *Parabacteroides*, *Anaerovorax*, *Cuneatibacter*, *Erysipelatoclostridium*, *Weissella*, *Holdemania*, *Klebsiella*, and *Enterococcus*. Spearman correlation analysis was used to analyze the associations among characteristic microbes, biochemical indexes, and fecal SCFAs. It was illustrated (Figure 9A) that the increased relative abundances of characteristic microbes of *Flavonifractor*, *Lactobacillus*, *Odoribacter* in DC group were positively related to the levels of GLP-1, HOMA-ISI, BW, isovaleric acid, isobutyric acid, acetic acid, *n*-butyric acid, IL-10, *n*-valeric acid were negatively associated with the TNF-α, LDL-c, HOMA-IRI, TC, TBA, GSP, FBG, TG, FFA, and IL-6 concentrations. In contrast to these three genera, the relative abundances of *Candidatus_Soleaferrea*, *Parabacteroides*, *Bacteroides*, *Weissella*, and others which enriched in SVP-H group showed an opposite correlation with biochemical indexes and SCFAs.

Moreover, the visualized network plot (Figure 9B) illustrated the strong correlation with a threshold of | *r*| > 0.6 and *p* < 0.01. On the one hand, *Flavonifractor* is one of the characteristic microbes in DC group, which was significantly positively related to the levels of TBA and TNF-α, and negatively correlated to BW level. It was reported that *Flavonifractor* belongs to Firmicutes and is positively associated with the level of ALT, AST, and TG in high-fat diet rats (50) and many circulating inflammatory markers (such as IL-1β, IL-6, and IL-21) in patients with end-stage kidney disease (51). The above results indicated that the increased abundances of *Flavonifractor* would regulate the levels of lipid metabolism-related indexes and inflammatory factors, and eventually lead to the occurrence of T2DM. On the other hand, *Odoribacter*, a genus belongs to Bacteroides phylum, also presents a higher abundance in DC group. And the relative abundance of *Odoribacter* was significantly negatively related to *n*-valeric acid and propionic acid levels, which is similar with a previous study (52). Besides, *Odoribacter* was reported to be enriched in hypercholesterolemic subjects (53) and highly abundant in *db/db* mice (54).

By contrast, oral SVP increased the relative abundances of *Weissella*, *Alloprevotella*, *Dubosiella*, *Erysipelatoclostridium*, etc. in T2DM mice. Of which, *Erysipelatoclostridium* and *Weissella* are important SCFAs-producing microorganisms, and the relative abundance of *Weissella* in this study was significantly positively associated with *n*-valeric acid, *n*-butyric acid, isovaleric acid, isobutyric acid whereas significantly negatively related to FFA level, which is consistent with many previous studies (55, 56). In addition, the relative abundance of *Alloprevotella* in this study was significantly positively associated with HOMA-ISI and negatively related to levels of FBG, GSP, TBA, TG, TC, and HOMA-IRI. It was reported that *Alloprevotella* has shown anti-inflammatory activities (57) and is negatively related to obesity and relative dyslipidemia (58), which is similar with our findings. Moreover, it was revealed the relative abundance of *Dubosiella* was significantly positively related to GLP-1, HOMA-ISI, BW and significantly negatively related to TG, HOMA-IRI, FBG, TC, LDL-c, TBA, and GSP levels, which is consistent with a previous study that a significant reduction of *Dubosiella* was found in T2DM mice. And it was shown that the *Dubosiella* abundance showed a positive relationship with levels of HDL-c, GSH-Px, T-AOC, SOD, and a negative relationship with TC, LDL-c, MDA and FBG levels (59). Additionally, the relative abundance of *Dubosiella* was also found made great contribution to the perturbations of lipid metabolism in the disease process (60). It was suggested that *Dubosiella* may contribute to alleviation of insulin resistance and lipid metabolism. Interestingly, it was reported that the *Erysipelatoclostridium* is one of the dominant intestinal genera in obesity mice (61), which was considered to be harmful to human health (62). However, the relative abundance of *Erysipelatoclostridium* in this study was significantly positively associated with propionic acid, *n*-valeric acid, acetic acid, isovaleric acid, *n*-butyric acid, isobutyric acid and significantly negatively related to FFA level. A previous study also showed *Erysipelatoclostridium* abundance was positively associated with the production of acetic acid and butyric acid (63). These findings demonstrated SVP could modulate the structure and function of intestinal microflora that produce more SCFAs to alleviate T2DM and its complications in T2DM mice.

## Conclusion

In the present research, characterization of SVP showed that it mainly contains small-molecular polysaccharides. And oral SVP strongly reduced the T2DM-induced abnormally elevated levels of FBG, GSP, OGTT, AUC of OGTT, HOMA-IRI, and markedly increased the levels of BW, GLP-1, HOMA-ISI in mice, indicating that SVP treatment could ameliorate the hyperglycemic state of T2DM. Oral administration of SVP in T2DM mice also could improve the diabetic complications of hyperlipidemia and inflammation response, indicating as lower concentrations of TC, TG, LDL-c, FFA, TBA, TNF-α, IL-6, and higher levels of IL-10 in serum. Especially, oral SVP strongly increased the concentrations of acetic acid, isobutyric acid, *n*-butyric acid, isovaleric acid, and *n*-valeric acid in feces of T2DM mice. Furthermore, 16S rRNA sequencing analysis demonstrated SVP treatment altered the structure of intestinal microflora and improve the gut imbalance, indicating as increased relative abundances of *Weissella*, *Alloprevotella*, *Dubosiella*, etc., and decreased relative abundances of *Lactobacillus*, *Flavonifractor*, *Odoribacter*, etc. The predicted metabolic functions of the intestinal microflora were also changed after SVP treatment in T2DM mice, such as higher levels of Citrate cycle, GABAergic synapse, Insulin signaling pathway, and lower levels of Purine metabolism, Taurine and hypotaurine metabolism, Starch and sucrose metabolism. These results illustrate that SVP has well potential to be a possible diabetes treatment.

## Data availability statement

The data presented in this study are deposited in the NCBI SRA BioProject repository, and the accession number is PRJNA867313.

## Ethics statement

The animal study was reviewed and approved by the Animal Ethics Committee of Fujian Agriculture and Forestry University.

## Author contributions

Z-RH conceived and designed the experiment and drafted the manuscript. Q-ZH and K-WC performed the experiments and collected the data. Z-FH and YL assisted with the interpretation of the data and checked the statistical analyses. R-BJ and BL provided the resources and reviewed the manuscript. All authors contributed to the article and approved the submitted version.
