# Supplementary material for: Sanghuangporus vaninii fruit body polysaccharide alleviates hyperglycemia and hyperlipidemia via modulating intestinal microflora in type 2 diabetic mice
Source: Front Nutr. 2022 Oct 17;9:1013466. doi: 10.3389/fnut.2022.1013466 (PMC9632624; doi:10.3389/fnut.2022.1013466)
Supplement: Supplementary file 1 [file Data_Sheet_1.docx]

Supplementary Material

# Supplementary Table 1. The monosaccharide composition analysis of SVP.

| **Sample** | **Monosaccharide composition (%)** | | | | | |
| --- | --- | --- | --- | --- | --- | --- |
| SVP | Man | Rha | GlcA | GalA | Gal | Arab |
|  | 35.23 | 6.45 | 2.33 | 9.64 | 16.13 | 28.94 |

# Supplementary Table 2. Relevant molecular parameters of SVP in MALLS analysis.

| **Peak No.** | **Molar mass moments (Da)** | | |  | **Polydispersity** | |  | **RMS radius moments (nm)** | | |
| --- | --- | --- | --- | --- | --- | --- | --- | --- | --- | --- |
|  | ***M_w_*** | ***M_n_*** | ***M_z_*** |  | ***M_w_/M_n_*** | ***M_z_/M_n_*** |  | ***R_w_*** | ***R_n_*** | ***R_z_*** |
| Peak 1 | 1.051 × 10^6^ (±1.777%) | 8.619 × 10^5^ (±1.771%) | 1.359 × 10^6^ (±3.991%) |  | 1.220  (±2.509%) | 1.577  (±4.367%) |  | 10.6  (±53.6%) | 9.6  (±65.7%) | 12.4  (±39.3%) |
| Peak 2 | 3.122 × 10^4^ (±2.997%) | 2.378 × 10^4^  (±2.881%) | 6.410 × 10^4^  (±5.868%) |  | 1.313  (±3.845%) | 2.695  (±6.537%) |  | 5.7  (±276.2%) | 5.5  (±319.3%) | 7.2  (±166.1%) |
| Peak 3 | 1.252 × 10^4^ (±13.399%) | 1.074 × 10^4^  (±11.02%) | 1.707 × 10^4^  (±36.553%) |  | 1.166  (±17.348%) | 1.590  (±38.178%) |  | 18.3  (±118.6%) | 17.9  (±117.4%) | 19.5  (±121.3%) |
| Peak 4 | 1.233 × 10^4^  (±42.501%) | 8.46 × 10^3^ (±43.209%) | 2.109 × 10^4^ (±92.822%) |  | 1.458  (±60.608%) | 2.493  (±102.386%) |  | - | - | - |

# Supplementary Table 3. The α-diversity of intestinal microflora in different groups.

| **Group** | **Shannon** | **Simpson** | **Chao1** | **ACE** |
| --- | --- | --- | --- | --- |
| NC | 5.02±0.35 | 0.88±0.04 | 520.15±49.77 | 526.23±49.26 |
| DC | 3.62±0.57 | 0.66±0.08 | 458.87±48.24 | 461.58±48.645 |
| MET | 4.87±1.03 | 0.86±0.13 | 451.72±6.18 | 457.99±3.60 |
| SVP-H | 5.02±1.02 | 0.89±0.06 | 486.82±160.37 | 466.34±112.99 |


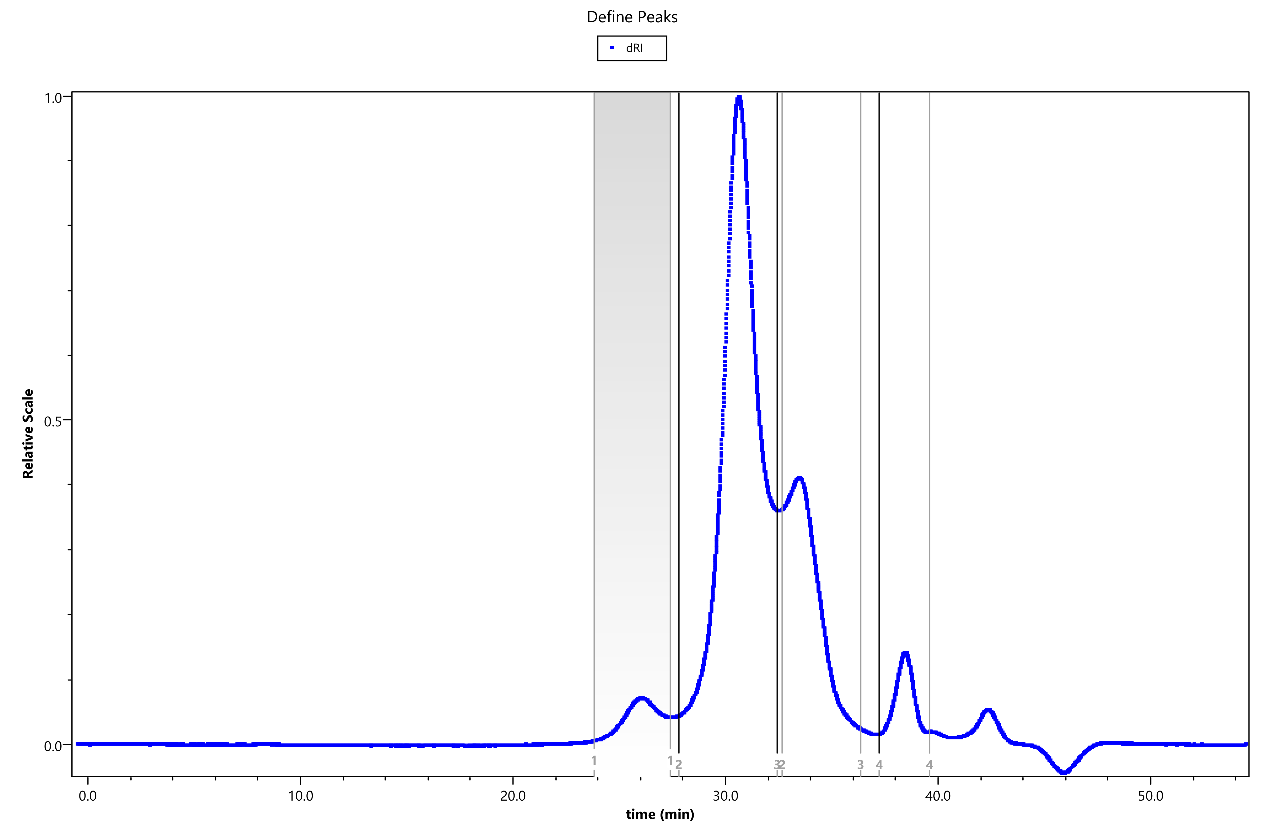


# Supplementary Figure 1. The homogeneity of SVP was analyzed by HPSEC-MALLS. Note: dRI: differential refractive index detector.


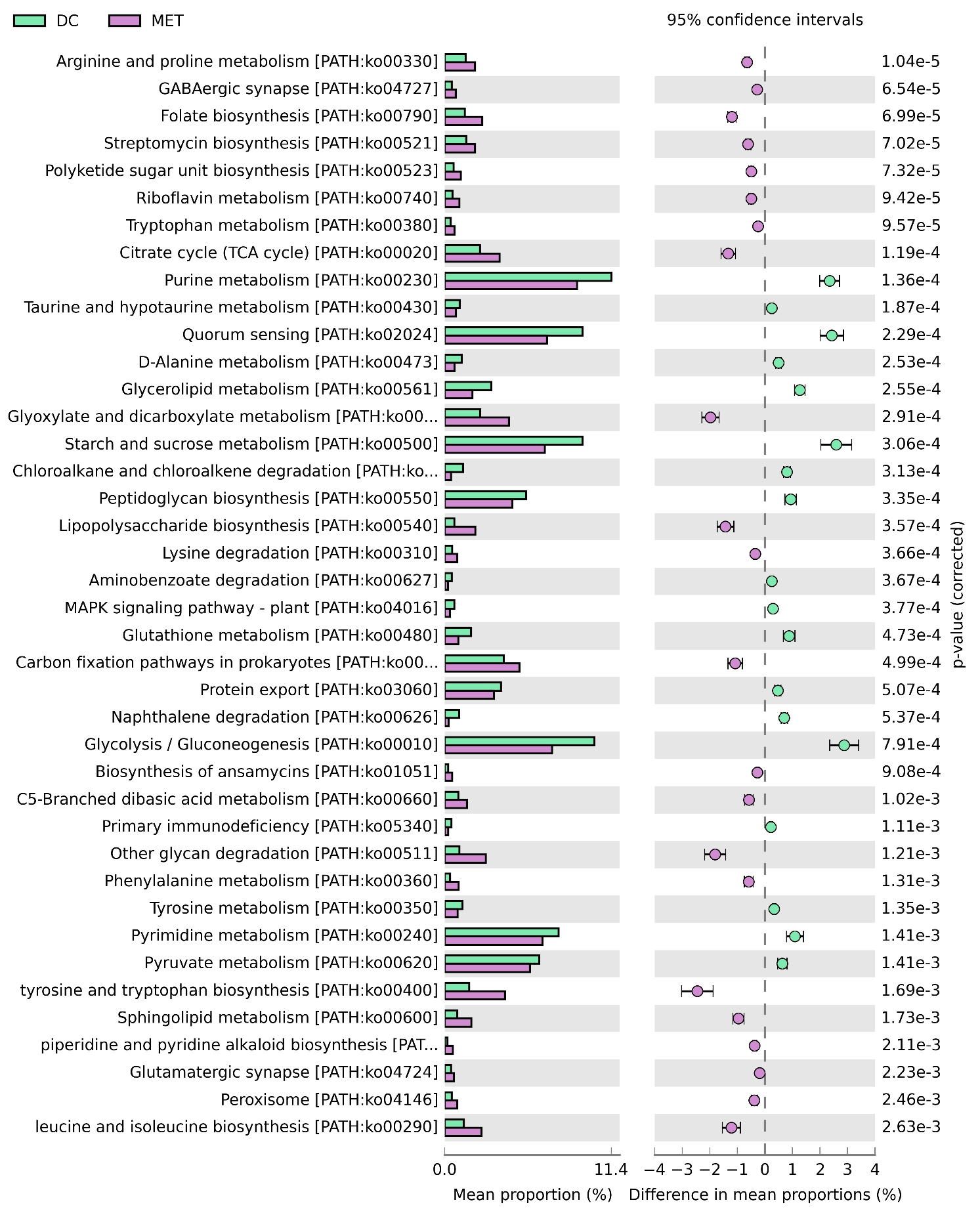


# Supplementary Figure 2. The differences of predicted function of the intestinal microbiota based on PICRUSt2 between DC group and MET group.
